# Integrated foam fractionation for heterologous rhamnolipid production with recombinant *Pseudomonas putida* in a bioreactor

**DOI:** 10.1186/s13568-016-0183-2

**Published:** 2016-02-09

**Authors:** Janina Beuker, Anke Steier, Andreas Wittgens, Frank Rosenau, Marius Henkel, Rudolf Hausmann

**Affiliations:** Institute of Food Science and Biotechnology, Department of Bioprocess Engineering (150k), University of Hohenheim, Fruwirthstr. 12, 70599 Stuttgart, Germany; Center for Peptide Pharmaceuticals, Ulm University, Meyerhofstr. 1, 89081 Ulm, Germany

**Keywords:** Foam fractionation, Heterologous rhamnolipid, *Pseudomonas putida*, Downstream processing, In situ product removal (ISPR), Biosurfactant

## Abstract

Heterologeous production of rhamnolipids in *Pseudomonas putida* is characterized by advantages of a non-pathogenic host and avoidance of the native quorum sensing regulation in *Pseudomonas aeruginosa.* Yet, downstream processing is a major problem in rhamnolipid production and increases in complexity at low rhamnolipid titers and when using chemical foam control. This leaves the necessity of a simple concentrating and purification method. Foam fractionation is an elegant method for in situ product removal when producing microbial surfactants. However, up to now in situ foam fractionation is nearly exclusively reported for the production of surfactin with *Bacillus subtilis*. So far no cultivation integrated foam fractionation process for rhamnolipid production has been reported. This is probably due to excessive bacterial foam enrichment in that system. In this article a simple integrated foam fractionation process is reported for heterologous rhamnolipid production in a bioreactor with easily manageable bacterial foam enrichments. Rhamnolipids were highly concentrated in the foam during the cultivation process with enrichment factors up to 200. The described process was evaluated at different pH, media compositions and temperatures. Foam fractionation processes were characterized by calculating procedural parameter including rhamnolipid and bacterial enrichment, rhamnolipid recovery, Y_X/S_, Y_P/X_, and specific as well as volumetric productivities. Comparing foam fractionation parameters of the rhamnolipid process with the surfactin process a high effectiveness of the integrated foam fractionation for rhamnolipid production was demonstrated.

**Electronic supplementary material:**

The online version of this article (doi:10.1186/s13568-016-0183-2) contains supplementary material, which is available to authorized users.

## Introduction

Consumer concern for renewable sources of products gained importance in the past. Microbially produced biosurfactants with their renewable raw material meet costumers requests. Rhamnolipids are one of the most intensively studied microbial produced biosurfactants. Rhamnolipids lower surface tension of water from 72 to 25–30 mN m^−1^ and exhibit CMCs (critical micelle concentration) as low as 10–200 mg L^−1^ (Lang and Wullbrandt 1999). Foam of rhamnolipids can exhibit gas contents from up to 99 % using rhamnolipid concentrations from 0.5, 1.0 and 1.5 % and display foam stabilities (time till half of the foam collapsed at atmospheric pressure and room temperature) of 17–41 min (Wang and Mulligan 2004).

Bergström et al. (1946) firstly described rhamnolipids and the structure of rhamnolipids was elucidated by Jarvis and Johnson (1949). In general, rhamnolipids contain one or two rhamnose moieties glycosidically bound to a lipid moiety made out of one, two or three β-hydroxy fatty acid chains which are in turn bound together through an ester bound (Abdel-Mawgoud et al. 2010). Depending on the amount of rhamnose moieties rhamnolipids are referred to as mono- or di- rhamnolipids.

Production of rhamnolipids is mainly described in the opportunistic pathogen *Pseudomonas aeruginosa* using shake flask, batch, fed-batch or continuous systems (Mulligan et al. 1989; Wei et al. 2005; Müller et al. 2010, 2012). Heterologeous production of rhamnolipids in *Pseudomonas putida* counteracts obstacles of strain pathogenicity and the native quorum sensing regulation in *P.aeruginosa* (Ochsner et al. 1995; Cha et al. 2008; Wittgens et al. 2011; Henkel et al. 2013, 2014). First heterologous rhamnolipid production was described by Ochsner et al. (1995) using *P.putida*, *Escherichia coli* and *Pseudomonas fluorescens* as recombinant production hosts. *P.putida* reached highest yields and productivities with 0.6 g L^−1^ and 25 mg L^−1^ h^−1^, respectively. Therefore, in this study the fully sequenced *P.putida* KT2440 was used exhibiting a non-pathogenic character, close relativity and therefore similar precursor capabilities to *P.aeruginosa* and availability of many established genetic tools like native and synthetic inducible promoter systems and different vector systems (Ochsner et al. 1995; Nelson et al. 2002; Loeschcke and Thies 2015). However, reported heterologous rhamnolipid production with maximal product concentration of 7.3 g L^−1^ (Cha et al. 2008) is by far not comparable to rhamnolipid production using *P.aeruginosa* wild type strains.

For rhamnolipid downstream processing several methods are reported in literature and well summarized by Mukherjee et al. (2006) and Heyd et al. (2008). Next to precipitation methods using either acid or ammonium sulfate followed by centrifugation (Zhang and Miller 1992; Déziel et al. 1999; Heyd et al. 2008) solvent extraction is a possible downstream processing method and may be combined with precipitation methods (Schenk et al. 1995). Following solvent extraction selective crystallization could be applied and/or chromatographic purification (Manso Pajarron et al. 1993) to obtain pure rhamnolipid crystals.

Next to solvent and precipitation methods also adsorption methods are used in rhamnolipid downstream processing (Reiling et al. 1986). However, using Amberlite 2 or 16 resins or wood activated carbon also requires solvents for rhamnolipid recovery (Dubey et al. 2005; Heyd et al. 2008).

Furthermore, anion exchange chromatography may be applied for downstream processing of rhamnolipids (Reiling et al. 1986). This method is based on the negative charge of rhamnolipids at high pH. However, anion exchange chromatography leads to a rhamnolipid mixture still containing some fatty acids as well as pigments.

Additionally membrane filtration may be used for rhamnolipid enrichment. The micelle building rhamnolipids can be retained using ultrafiltration with a membrane cutoff of 10 kDa (Mulligan and Gibbs 1990).

An elegant downstream processing method for in situ product concentrating and purification is foam fractionation. It was previously shown that simple cultivation integrated foam fractionation is an effective tool for biosurfactant production e.g. of surfactin using *Bacillussubtilis* (Chen et al. 2006; Willenbacher et al. 2014, 2015) or HFBII using *Saccharomyces cerevisiae* (Winterburn et al. 2011). The usability of foam fractionation in rhamnolipid purification and concentration in a cell free process was shown previously (Sarachat et al. 2010). However, cultivation integrated foam fractionation in rhamnolipid production was shown to be not feasible due to highly concentrated biomass in the foam leading to the necessity to develop solutions regarding cell retention e.g. using magnetic separation or cell recycling (Gruber 1991; Heyd et al. 2011; Küpper et al. 2013).

In this article a cultivation integrated foam fractionation process for rhamnolipids in a bioreactor using *P. putida* KT2440 as a heterologous production strain is described with low biomass enrichment in the foamate giving the opportunity to remove highly concentrated rhamnolipids from the cultivation broth in situ.

## Materials and methods

### Chemicals

All chemicals used in the current study were purchased from Carl Roth GmbH (Karlsruhe, Germany) if not stated otherwise.

### Microorganism and plasmid

A genetically engineered *P.putida* KT2440 strain producing mono-rhamnolipids was used in all foam fractionation experiments.

The genetic construct pSynPro8oT_rhlAB was obtained as follows. The vector backbone (pBBR-P^−^) was amplified from pBBR1MCS-3 (Kovach et al. 1995) without promoters, lac operator and *lacZ* gene and multiple cloning site using the forward primer AAAACTTAAGTGGGGTGCCTAATGAGTGAGCTAACTCAC and the reversed primer TTTAGATCTTAACCAATAGGCCGACTGCGATGAGTGG. The linear PCR product was phosphorylated and ligated.

To obtain a simple detection method for *rhlAB* transcription a *lov* gene, an oxygen independent fluorescing domain, was integrated in the vector construct. Therefore, a 745 bp fragment containing a *lov* gene, a *Mlu*I and *Ssp*I restriction site upstream of the *lov* gene, two flanking transcription terminators and restriction sites for *Bgl*II and *Bsp*TI (Term-*lov*-Term) was subcloned via *Bgl*II and *Bsp*TI into pBBR-P^−^ referred to as pTLT-vector. The Term-*lov*-Term fragment was designed as described below and produced by GeneArt AG (Regensburg, Germany). As terminators the BBa_B0015 variant available from iGEM (international genetically engineered machine competition) was used. Information for the *lov* gene and 20 bp upstream of the start codon was taken from a commercially available plasmid pGLOW-K^XN^-Bs2 (Evocatal GmbH, Düsseldorf, Germany). Additionally, two restriction sites (*Mlu*I and *Ssp*I) were integrated between the first terminator and the *lov* gene. A *Bgl*II and *Bsp*TI restriction site started and ended the fragment, respectively. The overall sequence of the Term-*lov*-Term fragment is provided as Additional file 1: Table S1.

For rhamnolipid transcription different new synthetic promoters (P_syn_) were developed following the strategy of Jensen and Hammer (1998). −35 and −10 regions were taken from the consensus sequence of σ^70^ promoters whereas spacer and flanking regions were randomized. Therefore, many promoters were generated harboring a sequence of 5′–NNNNN**TTGACA**NNNNNNNNNNNNNNNNN**TATAAT**NNNNNN–3′. To merge these promoters in front of the rhamnosyl transferase genes, forward primers were constructed containing DNA of the different promoter as well as a hybridized section for amplification of the *rhlAB* operon of *P.aeruginosa* PAO1 starting at the native transcription point 228 bp upstream of the *rhlA* start codon. After amplification the resulting PCR product was cut upstream of P_syn_ using *Sgs*I. The target vector pTLT was cut using *Mlu*I and *Ssp*I and ligated with the construct.

This strategy led to different rhamnolipid vectors, which were transformed in *P.putida* KT2440. Detection of rhamnolipid production proved difficult, because fluorescence of the *lov* gene product could not be detected in any colony. Therefore, rhamnolipid producer strains were indentified using the hemolytic activity of rhamnolipids detected via blood agar plates as described by Carrillo et al. (1996). Thereafter, rhamnolipid production efficiency was screened via an orcinol assay as described by Chandrasekaran and Bemiller (1980) and modified by Ochsner (1993). The highest rhamnolipid concentration as well as the highest rhamnolipid/OD amount could be detected for the plasmid pSynPro8_rhlAB. Also highest transcript amounts determined via real time PCR could be detected for pSynPro8_rhlAB with 0.1478 ng/50 ng and 0.0039 ng/50 ng for *rhlA* and *rhlB*, respectively.

To generate a stable vector, the terminator on the plasmid pSynPro8_rhlAB in front of the *rhlAB* gene was deleted. pSynPro8_rhlAB was cut using *Bgl*II and *Psi*I, ligated and transformed in *P.putida* KT2440 using electroporation as described in Troeschel et al. (2010). This plasmid was used in this study and is referred to as pSynPro8oT_rhlAB.

### Real time PCR

LB cultures were inoculated to OD_580_ 0.05 using an overnight culture and incubated at 30 °C for 24 h. Total RNA was isolated of 1 ml culture and cDNA was synthesized by reverse transcription. Afterwards, 50 ng cDNA was used for real time PCR and the amount of transcript was quantified using specific TaqMan^®^ probes (Applied Biosystems, Waltham, MA, USA). A PCR product of the synthetic *rhlAB*-*lov* operon was used as standard with its concentration photometrically determined beforehand.

## Culture conditions

### Media

Tetracycline was added to all media to an end concentration of 20 mg L^−1^.

For the first culture LB medium (5 g L^−1^ yeast extract (BD, Heidelberg), 10 g L^−1^ tryptone (BD, Heidelberg), 5 g L^−1^ NaCl; pH 7.0) was utilized. For seed culture either cultivation medium adapted from Wilms et al. (2001) using a phosophate buffer system (Wilms medium: 2.6 g L^−1^ K_2_HPO_4_, 0.65 g L^−1^ KH_2_PO_4_, 5 g L^−1^ (NH_4_)_2_SO_4_, 0.5 g L^−1^ NH_4_Cl, 2 g L^−1^ Na_2_SO_4_, 0.5 g L^−1^ MgSO_4_ ∙ 7 H_2_O, 35 g L^−1^glucose, 0.05 g L^−1^ Thiamin HCl, 3 mL L^−1^ trace element solution 1, pH 7.4; trace element solution 1: 0.18 g L^−1^ ZnSO_4_ ∙ 7 H_2_O, 0.16 g L^−1^ CuSO_4_ ∙ 5 H_2_O, 0.1 g L^−1^ MnSO_4_ ∙ H_2_O, 13.9 g L^−1^ FeCl_3_ ∙ 6 H_2_O, 10.05 g L^−1^ EDTA Titriplex III, 0.18 g L^−1^ CoCl_2_ ∙ 6 H_2_O, 0.662 g L^−1^ CaCl_2_ ∙ 2 H_2_O) or a second medium termed SupM (SupM medium: 4.4 g L^−1^ Na_2_HPO_4_ ∙ 2 H_2_O, 1.5 g L^−1^ KH_2_PO_4_, 1 g L^−1^ NH_4_Cl, 0.2 g L^−1^ MgSO_4_ ∙ 7 H_2_O, 0.02 g L^−1^ CaCl_2_ ∙ 2 H_2_O, 0.006 g L^−1^ FeCl_3_, 30 g L^−1^ glucose, 10 g L^−1^ yeast extract, 1 mL L^−1^ trace element solution 2, pH 6.8; trace element solution 2: 0.3 g L^−1^ H_3_BO_3_, 0.2 g L^−1^ CoCl_2_ ∙ 6 H_2_O, 0.1 g L^−1^ ZnSO_4_ ∙ 7 H_2_O, 0.03 g L^−1^ MnCl_2_ ∙ 4 H_2_O, 0.01 g L^−1^ CuCl_2_ ∙ 2 H_2_O, 0.03 g L^−1^ Na_2_MoO_4_ ∙ 2 H_2_O, 0.02 g L^−1^ NiCl_2_ ∙ 6 H_2_O) was applied. In the bioreactor cultivation either Wilms medium or a third medium termed ModR (22 g L^−1^ KH_2_PO_4_, 2.6 g L^−1^ (NH_4_)_2_HPO_4_, 1.4 g L^−1^ MgSO_4_ ∙ 7 H_2_O, 0.87 g L^−1^ citric acid, 0.01 g L^−1^ FeSO_4_ ∙ 7 H_2_O, 35 g L^−1^ glucose, 10 mL L^−1^ trace element solution 2, pH 6.8) was used.

### Preparation of seed culture

All shake flasks were inoculated in a shake incubator chamber (Multitron II, HT Infors, Bottmingen, Switzerland) at 30 °C and 120 rpm. First 25 mL LB in a 100 mL baffled shake flask were inoculated with 50 µL from a glycerol stock solution of *P. putida* KT2440 pSynPro8oT_rhlAB and incubated for 24 h. Seed cultures contained 100 mL Wilms or SupM medium in a 1 L baffled shake flask inoculated with 1 mL from the 24 h LB culture and incubated for 12 h.

### Bioreactor cultivations

**Fig. 1:**
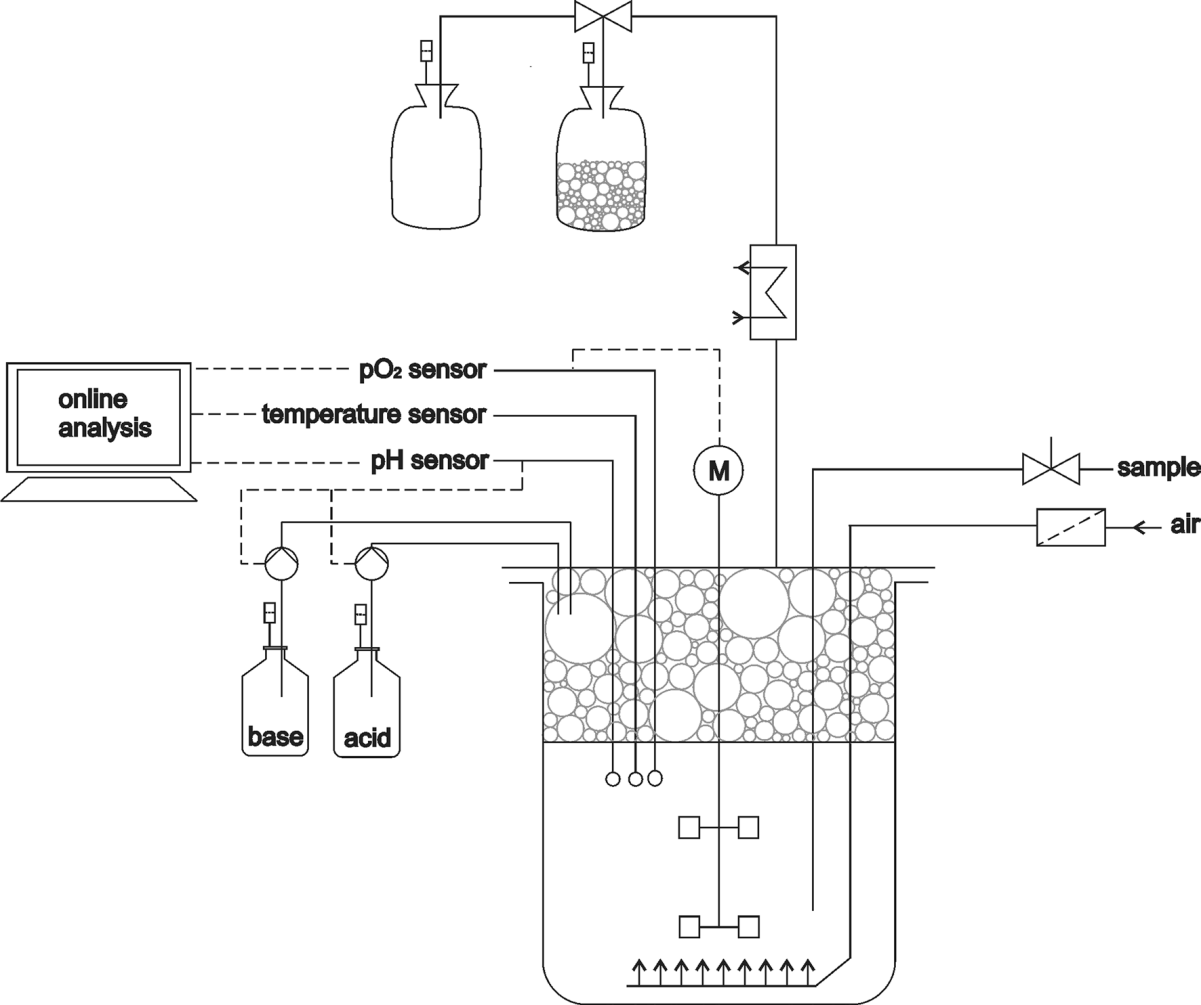
Setup for integrated foam fractionation in a bioreactor. Foam is generated during cultivation process in the bioreactor and channeled through the off gas cooler into cooled exchangeable foam bags

All bioreactor cultivations were carried out as duplicates. The bioreactor setup was similar as described in Willenbacher et al. (2014) and illustrated in Fig. 1. The bioreactor (Minifors, HT Infors, Bottmingen, Switzerland) was equipped with an integrated pH, temperature and aeration control system. Aeration was set at 0.067 vvm and pO_2_ was controlled at 13 % via stirring rate starting with a minimum of 300 rpm. Bioreactors were inoculated with the 12 h seed culture to a final OD of 0.5 but no more than 10 % *v*/*v*. Since foam fractionation was applied, generated foam was channeled through the exhaust cooler and the different fractions were collected in cooled interchangeable bags. Bioreactor cultivations were terminated upon glucose depletion in the bioreactor.

In Wilms medium inoculation of the bioreactor was conducted using 12 h Wilms seed culture. During bioreactor cultivation temperature was held constant at 33 °C and pH was adjusted to 7.4 via 4 M H_3_PO_4_ or 4 M NaOH.

In ModR medium inoculation of the bioreactor was conducted using 12 h SupM seed culture. During bioreactor cultivation temperature was held constant at 30 °C. Due to pH controlling to 6.8 via 1 M H_2_SO_4_ or 19 % NH_4_OH ammonium concentration was also held constant.

## Analytical methods

### Sampling and processing

At each sampling point foam fractions were collected, samples were taken from the bioreactor and the collapsed foam fractions (foamate) and foamate volume was determined. Bioreactor and foamate samples were treated equally.

For biomass determination OD_580_ was measured and divided by a OD_580_/biomass correlation factor of 3.25 OD_580_/(g L^−1^).

The remaining sample was centrifuged (13,200 rpm, 15 min) to gain cell free supernatant for rhamnolipid, glucose and ammonium detection.

Rhamnolipid detection was performed as described by Schenk et al. (1995) with minor adjustments. Part of the liquid phase was acidified 1:100 (*v*/*v*) by phosphoric acid and rhamnolipids were extracted twice with 1.25 vol. ethyl acetate. For rhamnolipid measurement appropriate amount of this ethyl acetate extract was evaporated. Rhamnolipids were resolved in acetonitrile and derivatized for 90 min at 1400 rpm and 60 °C using a 1:1 mixture of 40 mM bromphenacylbromid and 20 mM tri-ethyl-ammonium/-amin. Detection of rhamnolipids was performed using a HPLC device (Agilent 1100 Series, Agilent, Waldbronn, Germany) equipped with a 15 cm reversed phase column (Supelcosil^®^ LC-18, Supelco, Deisenhofen, Germany) at 30 °C. The mobile phase was composed of 100 % methanol and ultrapure water. For rhamnolipid detection a gradient was applied. During the first 17 min methanol concentration was increased to 100 % starting at 80 %. This methanol concentration was held for 8 min and decreased to 80 % during the next 5 min. Rhamnolipids were detected at a wave length of 254 nm at 30 °C. For calibration standard solutions of rhamnolipid in acetonitrile were used.

The concentration of glucose and ammonium were detected from the aqueous phase of samples using glucose (Cat. no. 10 716 251 035, R-Biopharm AG, Darmstadt, Germany) and ammonium (1.14752.001, Merck KGaA, Darmstadt, Germany) assay kits, respectively, according to the manufacturers’ instructions.

### Data analysis

To analyze and characterize the different bioreactor cultivations total combined masses of biomass, rhamnolipid and glucose in the bioreactor and the integral foam fractions were calculated and defined as “overall values”. These overall values were fitted using a logistic equation with four parameters in a scientific data analysis and graphing software (Sigma Plot 12.5, Systat, San Jose, USA).

1 $$y = y_{0} + \frac{a}{{1 + \left( {\frac{x}{{x_{0} }}} \right)^{b} }}$$

With these curves Y_X/S_ [g g^−1^], Y_P/X_ [g∙g^−1^], µ [h^−1^], spec. q_RL_ [mg g^−1^ h^−1^] and vol. q_RL_ [mg l^−1^ h^−1^] were calculated. Bacterial and rhamnolipid enrichment and rhamnolipid recovery [%] were determined using measurement data.

Y_X/S_ and Y_P/X_ were determined in an integral manner using fitted glucose, biomass and rhamnolipid masses of the overall process at the time point of glucose depletion in the bioreactor.

2 $$Y_{X/S} = \frac{{\rm\Delta {\it m}_{{\it X}} }}{{\rm\Delta {\it m}_{{\it Glu}} }};\quad Y_{P/X} = \frac{{\rm\Delta {\it m}_{{\it P}} }}{{\rm\Delta {\it m}_{{\it X}} }}$$

Growth rates were calculated in a differential manner using fits of the overall biomass.

3 $$\mu (t_{2} ) = \frac{{ln\left( {m_{X\_t2}} / {m_{X\_t1}} \right)}}{{\Delta {\it t}({\it t}_{{\it 2}} ,{\it t}_{{\it 1}} )}}$$

Differential and integral specific productivities were calculated using fitted biomass and rhamnolipid masses of the overall process. However, in differential calculations the mean of biomass before and at the specific time point was considered whereas in integral calculations the overall biomass produced was used.

4 $$dif. spec. q_{RL} \left( {t_{2} } \right) = \frac{{\rm\Delta {\it m}_{{\it RL}} \left( {{\it t}_{2} ,{\it t}_{1} } \right)}}{{\emptyset {\it m}_{{\it X}} \left( {{\it t}_{1} ,{\it t}_{2} } \right) \times \rm\Delta {\it t}\left( {{\it t}_{2} ,{\it t}_{1} } \right)}};int. spec. q_{RL} = \frac{{\rm\Delta {\it m}_{{\it RL}} }}{{\rm\Delta {\it m}_{{\it X}} \times \rm\Delta {\it t}}}$$

Integral volumetric productivities were calculated using fitted rhamnolipid masses of the overall process.

5 $$vol. q_{RL} = \frac{{\rm\Delta {\it m}_{{\it RL}} }}{{{\it V}_{{\it total}} \times \rm\Delta {\it t}}}$$

Bacterial and rhamnolipid enrichments were calculated in a differential manner using measurements of bacterial and rhamnolipid concentration in foamate and bioreactor. The concentration of a component in the foamate was divided by its mean concentration in the bioreactor at sampling and previous sampling.

6 $$enrichment(t_{2} ) = \frac{{c_{i foam} (t_{2} )}}{{\emptyset c_{i fermenter} (t_{1} ,t_{2} )}}$$

Rhamnolipid recovery was calculated in an integral manner using measured rhamnolipid masses in foamate and bioreactor.

7 $$recovery = \frac{{m_{RL foam} }}{{m_{RL foam} + m_{RL fermenter} }}$$

## Results

### Time courses of overall biomass, rhamnolipid and glucose during bioreactor cultivation

**Fig. 2:**
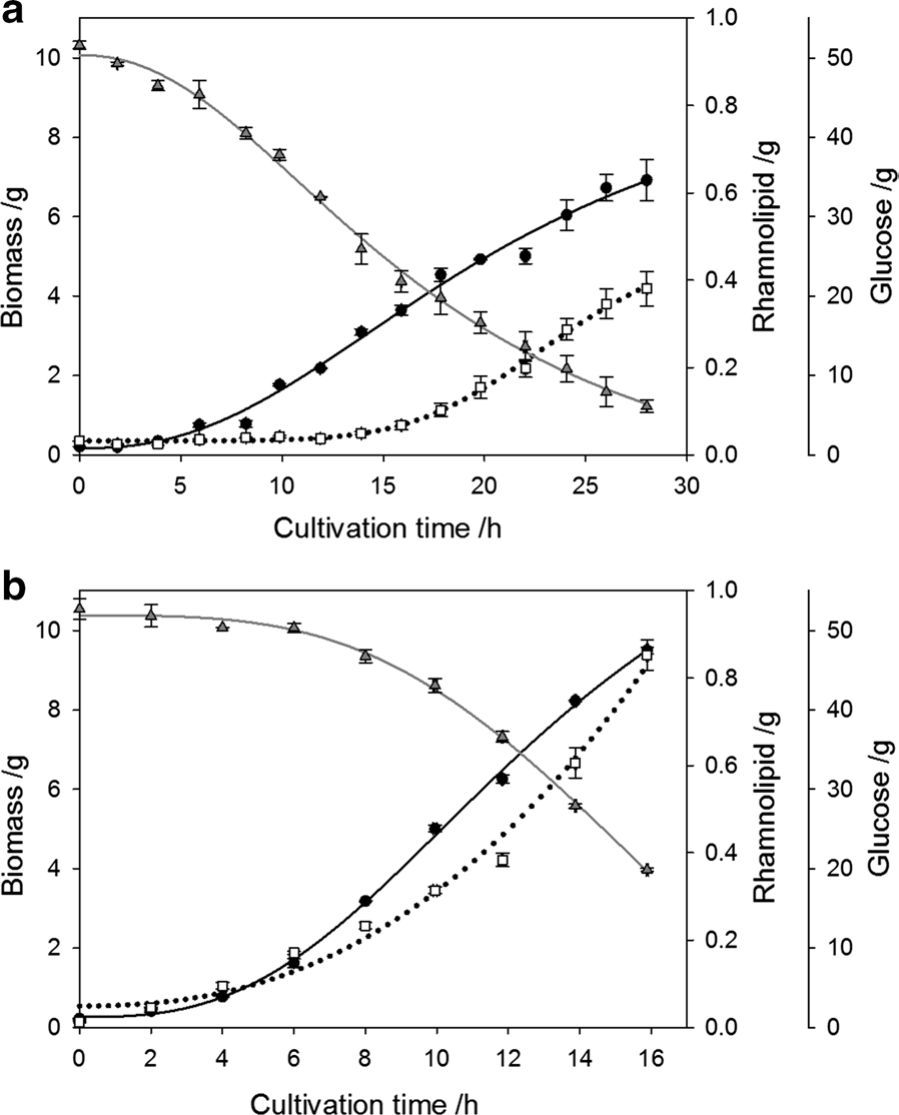
Time course of overall biomass, glucose and rhamnolipid masses (sum of bioreactor and integral foam fractions) during foam fractionation process. **a** shows results of bioreactor cultivations using Wilms medium setup, **b** shows results of bioreactor cultivations using ModR medium setup. The values for biomass (*black circles*), glucose (*grey triangles*) and rhamnolipid (*blank squares*) are given as mean values of two bioreactor cultivations. *Dotted*, *solid black* and *solid grey lines* represent the logistic fit functions of the rhamnolipid, biomass and glucose time course, respectively based on Eq. 1

Time courses of the overall (sum of bioreactor and integral foam fractions) generated biomass and rhamnolipid and consumed glucose are depicted in Fig. 2. Using Wilms medium setup a maximal overall biomass of 6.9 ± 0.5 g and maximal overall rhamnolipid mass of 0.38 ± 0.04 g was reached at the end of bioreactor cultivation after 28 h. However, rhamnolipid production did not start until 16 h. Until the end of bioreactor cultivation glucose was depleted in the bioreactor medium and 5.23 ± 0.04 g was removed by foaming.

Using ModR medium setup the cultivation till glucose depletion in the bioreactor took 16 h and 16.1 ± 0.2 g glucose was removed by foaming. Maximal overall biomass of 9.5 ± 0.1 g and maximal overall rhamnolipid masses of 0.85 ± 0.03 g were reached at the end of bioreactor cultivation.

In general, bioreactor cultivation using Wilms medium setup took 12 h longer. Additionally just 73 % of the biomass and 45 % of the rhamnolipid was produced using Wilms medium setup compared to ModR medium setup.

### Time course of bacterial and rhamnolipid enrichment and rhamnolipid recovery

**Fig. 3:**
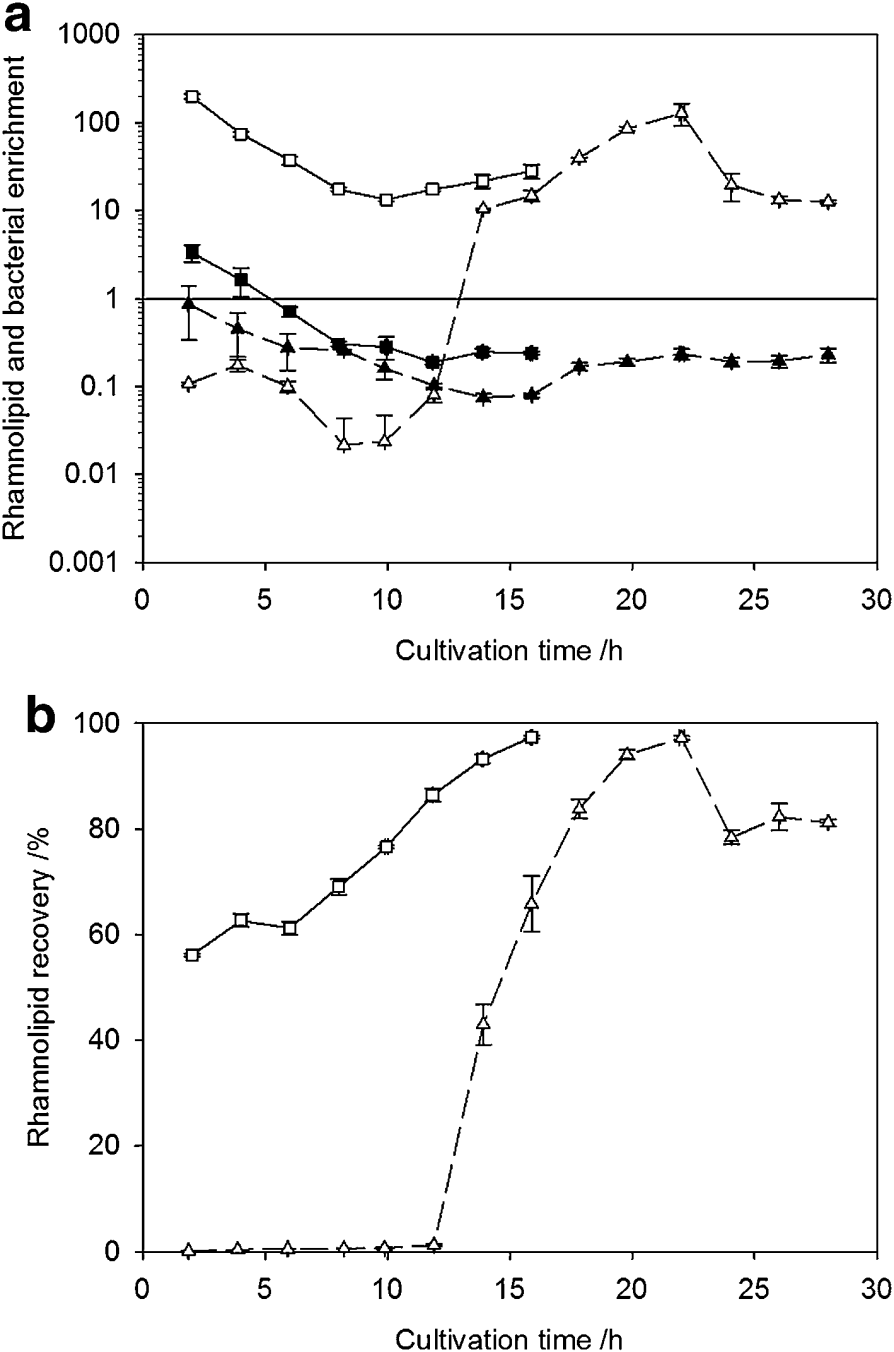
Time course of differential bacterial and rhamnolipid enrichment and integral rhamnolipid recovery during foam fractionation process. **a** shows rhamnolipid (*blank*) and bacterial (*black*) enrichments using Wilms (*triangles*) and ModR (*squares*) medium setup referring to a logarithmic axis. The values for rhamnolipid and bacterial enrichment were calculated as depicted in Eq. 6, **b** shows rhamnolipid recovery using Wilms (*triangles*) and ModR (*squares*) medium setup calculated according to Eq. 7

Rhamnolipid recovery and bacterial and rhamnolipid enrichment were calculated using Eqs. 6 and 7, respectively and are depicted in Fig. 3.

Using Wilms medium setup biomass enrichment was constantly lower 1 representing a lower biomass concentration in the foamate compared to the bioreactor. Rhamnolipid enrichment also started at values lower 1 but increased upon rhamnolipid production after 16 h to values up to 129 to decrease till the end of bioreactor cultivation to values around 15. Therefore upon rhamnolipids production, rhamnolipids were constantly concentrated in and removed by the foam. Rhamnolipid recovery started at low values but increased upon rhamnolipid production to values up to 97 % and a final value of 81 %.

Using ModR medium setup biomass enrichment started at values slightly higher 1 but decreased immediately to values lower 1 representing a lower biomass concentration in the foamate compared to the bioreactor. Rhamnolipid enrichment started at high values of up to 198 and decreased over time to values around 20 with a slightly increasing trend in the end. Therefore, rhamnolipids were constantly highly concentrated in the foamate. Rhamnolipid recovery started at 56 % and increased over time to 97 %.

### Process parameters of foam fractionations in different setups using* P. putida*—rhamnolipid system and comparison to* B. subtilis*—surfactin system

Process parameters of the different *P.putida*—rhamnolipid setups are summarized in Table 1. Furthermore, to compare foam fractionation of two different biosurfactant systems the *P.putida*—rhamnolipid system is compared to the *B.subtilis*—surfactin system. For this the mean of different process parameters of all *B.* *subtilis* strains displayed in Willenbacher et al. (2014) were calculated and depicted in Table 1.

**Table 1:** Process parameter of different media setups of the *P. putida*—rhamnolipid system and *B. subtilis*—surfactin system

	*P. putida*—rhamnolipid in Wilms		*P. putida*—rhamnolipid in ModR		*B. subtilis*—surfactin	
Overall Y_P/X_ (g/g)	0.06	±0.01	0.10	±0.01	0.15	±0.07
Overall Y_X/S_ (g/g)	0.16	±0.02	0.26	±0.02	0.24	±0.06
Max. q_Product_ [mg/(g∙h)]	5.90	±0.29	24.71	±0.69	45.00	±26.93
Integral q_Product_ [mg/(g∙h)]	1.89	±0.44	5.57	±0.39	4.37	±1.98
Max. vol. q_Product_ [mg/(L∙h)]	8.70	±1.16	38.09	±2.12	46.25	±35.68
Overall product recovery (%)	83.39	±1.51	97.38	±0.33	85.35	±13.19
Max. product enrichment	128.54	±36.54	197.98	±13.42	80.68	±39.73
Mean bact. enrichment	0.25	±0.07	0.86	±0.19	0.79	±0.45
Max. product conc. in foam (g/L)	1.04	±0.04	2.15	±0.04	2.35	±0.76
Product recovered in foam (g)	0.30	±0.02	0.83	±0.03	0.53	±0.35

The mean of different process parameters of all *B. subtilis*—surfactin systems is depicted (Willenbacher et al. 2014). In *P. putida*—rhamnolipid systems Y_P/X_, Y_X/S_, max. q_Product_, integral q_Product_ and max. vol q_Product_ were calculated using logistic fit data whereas all other parameter were calculated using measured data

Comparing process parameter of the two different media, pH and temperature setups for rhamnolipid production bioreactor cultivations using ModR medium setup reached higher values in all process parameter. High differences could be detected in productivities. The maximal specific as well as the volumetric rhamnolipid productivity using the ModR medium setup was 4.2 and 4.4 times higher, respectively. Also integral specific rhamnolipid productivity was 2.9 times higher using ModR medium setup compared to Wilms medium setup. Additionally, maximal product concentration in the foamate and product recovered in the foamate using ModR medium setup was 2.1 and 2.8 times higher than using Wilms medium setup, respectively. Furthermore, mean bacterial enrichment was 3.5 times higher using ModR medium setup than using Wilms medium setup. However, in both setups bacterial enrichments were lower than 1 accounting for lower biomass concentration in the foam than in the bioreactor.

Comparing the two different foam fractionation systems it becomes evident that all process parameters are quite similar in the *B.subtilis*—surfactin and in the *P.putida*—rhamnolipid system using ModR medium setup. However, differences could be detected in maximal product enrichment with higher enrichments using the *P.putida* system applying ModR medium setup (2.5 times higher).

## Discussion

### Production kinetics

Comparing the two different media, pH values and temperature conditions used in this study different rhamnolipid production kinetics could be detected. Whereas rhamnolipid production is growth associated using ModR medium conditions, it starts not until 16 h using Wilms medium conditions even though the same organism with the same plasmid is used in both cultivations.

Guerra-Santos et al. (1984, 1986) showed that temperature, pH and medium composition have an influence on rhamnolipid production in *P.aeruginosa*. Temperatures of 32–34 °C and pH values of 6.2–6.4 were advantageous for rhamnolipid production. The lower pH of 6.8 using ModR medium conditions could therefore be one of the reasons for increased rhamnolipid production. However, Wilms medium conditions would favor rhamnolipid production regarding temperature.

Also, differences in kinetics may be caused by medium compositions. The cultivations in this study were carried out in batch mode and therefore medium compounds were consumed over time. Guerra-Santos et al. (1984, 1986) studied rhamnolipid production in *P.aeruginosa* depending on media compositions. Elements with a major effect on rhamnolipid production were iron as well as calcium, nitrogen, magnesium and phosphor with an increased rhamnolipid production upon iron limitation (27.5 10^−3^ mg L^−1^), a C–to–N ratio of 18, optimal C–to–Mg ratio of 364 or higher (MgSO_4_ ∙ 7 H_2_O concentrations below 0.2 g L^−1^) and a surplus of phosphor with a C–to–P ratio of 16. Additionally, sodium, potassium, and calcium reduction caused higher rhamnolipid production. Persson et al. (1990) also studied the influence of medium compositions on biosurfactant production in *P.fluorescens*. In their studies they elucidated a major effect of iron on biosurfactant production whereas the carbon to nitrogen ratio as well as the phosphor concentration did not influence biosurfactant production. Taking these results into account differences in rhamnolipid production kinetics could be caused by different iron concentrations. In ModR medium the iron concentration (2 mg L^−1^) is more than four times lower than in Wilms medium. Therefore, fast iron limitation could induce rhamnolipid production using ModR medium whereas high iron concentrations could influence a delay of rhamnolipid production using Wilms medium. Additionally, high phosphor concentrations (9 × higher) and calcium deficiency in ModR could also lead to beneficial conditions for rhamnolipid production. However, potassium and magnesium concentration are higher in ModR medium than in Wilms medium having a negative effect on rhamnolipid production in *P.aeruginosa*. Concluding, the main reason of different rhamnolipid production kinetics using different cultivation conditions and media is suspected to be caused by differences in medium compositions or cultivation conditions.

### Effective foam fractionation

The main finding under the experimental setup in this article was unexpected low biomass enrichment in the foamate in contrast to other statements in the literature (Gruber 1991; Heyd et al. 2008; Küpper et al. 2013).

This led to an unforeseeable effective method to produce and in situ concentrate rhamnolipids via simple cultivation integrated foam fractionation using the heterologous production strain *P.putida KT2440* containing a plasmid for mono-rhamnolipid production in a bioreactor.

Highly enriched biomass as described by Küpper et al. (2013) and Gruber (1991) could be the effect of particle flotation. It was shown before that a negative charge of particles could be reversed using multivalent anionic ions (e.g. Mg^2+^) and flotated using anionic surfactants (Somasundaran 1975). If negatively charged bacteria (Hubbuch et al. 2006) are also seen as particles their charge could be reversed by Mg^2+^ ions present in the cultivation medium and they could be flotated by negatively charged produced rhamnolipids. Grieves and Wang (1967a, b) support this thesis with a couple of experiments. Using cationic surfactants *P.fluorescens* as well as *B.subtilis* var *niger* and other bacteria suspended in distilled water could be readily removed by foaming (Grieves and Wang 1967b). However, bacterial foam enrichment reduced dramatically in both cases using the same setup but adding Mg^2+^ or other bivalently charged ions to the media with a larger impact on the enrichment of *P.fluorescens* than of *B.subtilis* var *niger* (Grieves and Wang 1967a). High influence of Mg^2+^ on the flotability of *Pseudomonas* could be one of the reasons why also *P.aeruginosa* and *P.putida* in the studies of Küpper et al. (2013) and Gruber (1991) were enriched in the foam using rhamnolipid production systems whereas in the surfactin systems little bacterial enrichment occurred (Willenbacher et al. 2014). Contrary, in this study *P.putida* KT2440 pSynPro8oT_rhlAB was not enriched in the foam suggesting that the combination of bacteria type and medium have an influence on bacterial foam adhesion.

Gruber (1991) used wild type strain *P. aeruginosa* DSM2659 producing mono- and di-rhamnolipids. He showed that bacterial enrichment was higher than rhamnolipid enrichment independent of the retention times of the foam in the foam column with a final bacterial enrichment of 3.4 and rhamnolipid enrichment of 2 at a retention time of about 40 min. Comparing Gruber’s finding with results shown in this article suggests that either the producer strain or media could cause different bacterial enrichments in the foamate.

Astonishingly, Küpper et al. (2013) used a very similar system to the one used in this study but also described high bacterial enrichments in the foamate of up to 3. Küpper et al. (2013) also exploited genetically engineered *P.putida* producing just mono-rhamnolipids but used LB medium as production medium. Furthermore, Küpper et al. (2013) did not state the exact organism and plasmid used in his studies. As production host the authors could have used either *P.putida* KT2440 or *P.putida* KT42C1, a rifampicin resistant (*P.putida* KT2442) and polyhydroxyalcanoates (PHA) negative (*P.putida* KT42C1) mutant of *P.putida* KT2440 (de Eugenio et al. 2010; Wittgens et al. 2011; Goldstein 2014). Follonier et al. (2011) examined differences of PHA building in KT2440 and KT2442 and questioned transferability of results between KT2440 and KT2442. Therefore, it could be suggested that the used production hosts of Küpper et al. (2013) could exhibit differences to the one used in this article. As plasmids Küpper et al. (2013) could have used either pVLT31_rhlAB or pVLT33_rhlAB being the same IPTG inducible construct but tetracycline and kanamycin resistant, respectively (de Lorenzo et al. 1993) with genomic information for *rhlA* and *rhlB* taken from *P.aeruginosa* PAO1 (Wittgens et al. 2011). Differences in the plasmids used by Küpper et al. (2013) and the one used in this article are therefore given either in antibiotic resistances or by different plasmidic backbones.

In summary, differences in bacterial attachments to the foam are suggested to be based on variations in the outer membrane compositions or membrane characteristics in specific media. The low enrichment of the bacteria used in this article is little affected by pH, temperature or defined medium composition. However, taking former literature into account suggests that complex medium could have an effect on bacterial enrichment. Different membrane compositions or membrane characteristics may be caused by differences of strains, different antibiotic resistances or other dissimilarities related to the plasmidic backbone. Yet, a distinct reason for differences in bacterial foam adhesion could not be determined and should be investigated in further detail.

However, taking advantage of the non adhesiveness of the cells this article presents an effective simple cultivation integrated foam fractionation method to produce and in situ concentrate rhamnolipids using a heterologous production strain independent of pH, temperature and defined medium composition with little cell accumulation in the foam.

## Additional file

10.1186/s13568-016-0183-2 Sequence of the synthetic oligonucleotide Term-lov-Term.
