# Supplementary material for: Integrated foam fractionation for heterologous rhamnolipid production with recombinant Pseudomonas putida in a bioreactor
Source: AMB Express. 2016 Feb 9;6:11. doi: 10.1186/s13568-016-0183-2 (PMC4747948; doi:10.1186/s13568-016-0183-2)
Supplement: Supplementary file 1 — 10.1186/s13568-016-0183-2 Sequence of the synthetic oligonucleotide Term-lov-Term. [file 13568_2016_183_MOESM1_ESM.pdf]

Table 1 Sequence of the synthetic oligonucleotide Term-lov-Term

AAAAGATCTCCAGGCATCAAATAAAACGAAAGGCTCAGTCGAAAGACTGGGCCTTTCGTTTTATCTG<br>TTGTTTGTCGGTGAACGCTCTCTACTAGAGTCACACTGGCTCACCTTCGGGTGGGCCTTTCTGCGTTT<br>ATAACGCGTAATAAAATATTGGCGCGCCTTTAAGAAGGAGATATACATATGGCGTCGTTCCAGTCGT<br>TCGGCATCCCGGGCCAGCTGGAAGTCATCAAGAAGGCGCTGGATCACGTGCGCGTCGGCGTGGTCA<br>TCACCGATCCCGCGCTGGAAGATAACCCGATCGTCTACGTGAACCAGGGCTTCGTGCAGATGACCGG<br>CTACGAGACCGAGGAAATCCTGGGCAAGAACGCGCGCTTCCTCCAGGGGAAGCACACCGATCCGGC<br>GGAAGTGGACAACATCCGCACCGCGCTGCAAAATAAAGAACCGGTCACCGTGCAGATCCAGAACTA<br>CAAGAAGGACGGCACGATGTTCTGGAACGAACTGAACATCGATCCGATGGAAATCGAGGATAAGAC<br>GTATTTCGTCGGCATCCAGAACGACATCACCAAGCAGAAGGAATATGAAAAGCTGCTCGAGCACCAC<br>CACCACCACCACTGAGGCGCGCCCCAGGCATCAAATAAAACGAAAGGCTCAGTCGAAAGACTGGGC<br>CTTTCGTTTTATCTGTTGTTTGTCGGTGAACGCTCTCTACTAGAGTCACACTGGCTCACCTTCGGGTGG<br>GCCTTTCTGCGTTTATACTTAAGAAAA
